# The Macro-Flora from the Middle–Late Cenomanian Paleontological Area of Algora (Guadalajara, Central Spain) and Its Paleobiogeographical and Paleoenvironmental Implications

**DOI:** 10.3390/biology15030250

**Published:** 2026-01-29

**Authors:** Luis M. Sender, Carlos A. Bueno-Cebollada, Adán Pérez-García

**Affiliations:** 1 Fundación Conjunto Paleontológico de Teruel-Dinópolis/Museo Aragonés de Paleontología, Avenida Sagunto s/n, 44002 Teruel, Spain; 2 Departamento de Geodinámica, Estratigrafía y Paleontología, Facultad de Ciencias Geológicas, Universidad Complutense de Madrid, c/José Antonio Nováis 12, 28040 Madrid, Spain; c.bueno@igme.es; 3 Grupo de Biología Evolutiva, Departamento de Física Matemática y de Fluidos, Facultad de Ciencias, Universidad Nacional de Educación a Distancia (UNED), Avda. Esparta s/n, 28232 Las Rozas de Madrid, Spain; a.perez.garcia@ccia.uned.es

**Keywords:** fossil plants, upper cretaceous, Tethys realm, fossil gymnosperms, cretaceous coastal environments

## Abstract

The middle–late Cenomanian paleontological area of Algora represents the main concentration of vertebrate remains from the basal Late Cretaceous for southwestern Europe. An unpublished macro-plant assemblage is studied here, being recognized as composed of ferns, conifers, and various other types of gymnosperms and angiosperms, constituting the first well-defined reference of a Cenomanian macro-flora in Spain. Comparison of this assemblage with other coeval ones from the western Tethys region suggests a possible influence of both Central European Laurasian plant elements and those from northern Gondwana (originating in North Africa and the Middle East) in this area of the Iberian Plate, a key region for understanding the dispersal of fauna and flora during the early Late Cretaceous.

## Simple Summary

Macroscopic plant remains from the beggining of the Late Cretaceous are not very abundant in the Iberian Peninsula, and there are also very few records of them in southwestern Europe. Therefore, the plant macro-remains assemblage from the Algora vertebrate site in central Spain, dating back approximately 96 million years, provides very important information about the types of plants and environments in which dinosaurs and other vertebrates lived in southern Europe during the early Late Cretaceous.

## 1. Introduction

The Cenomanian site of Algora presents the largest concentration of vertebrates identified in the southwestern European record. Reference [1] updated the faunal information on this locality, recognizing the presence of eight taxa: a lepisosteoid fish corresponding to *Obaichthys africanus*; two turtles, one of them being a basal terrestrial form (the helochelydrid aff. *Plastremys lata*) and the other a pleurodiran bothremydid described based on several fossil remains from this locality (*Algorachelus peregrina*, being the most abundant vertebrate in Algora); a plesiosaur identified as an indeterminate elasmosaurid; two indeterminate crocodyliforms, referred to as a non-eusuchian neosuchian and an eusuchian; and two dinosaurs, represented by an indeterminate lithostrotian titanosaur and a theropod identified as cf. Abelisauridae [1,2,3,4,5,6,7,8,9,10]. Among the unpublished finds are new remains of Osteichthyes that allow the recognition of the presence of several taxa; the identification of chondrichthyan remains; and the discovery of a skeleton of a relatively large cryptodiran sea turtle [9]. Furthermore, the presence of Squamata has been recently recognized [11,12], a new form of Pythonomorpha having been defined: *Carentonosaurus algorensis* [13]. Regarding the plant remains, although they are abundant and diverse in this Spanish locality, there are no previous studies to date, and only the presence of gymnosperms based on logs with ferruginized xylem and cycadaceans by a supposed external impression of a trunk has been previously reported [14]. In this work, we present the description of components of the macro-paleobotanical assemblage found at the moment in this site, comparing it with coeval floras and infering both paleoenvironmental and paleobiogeographical implications for the Tethysian realm during Cenomanian times.

## 2. Materials and Methods

**Figure 1:**
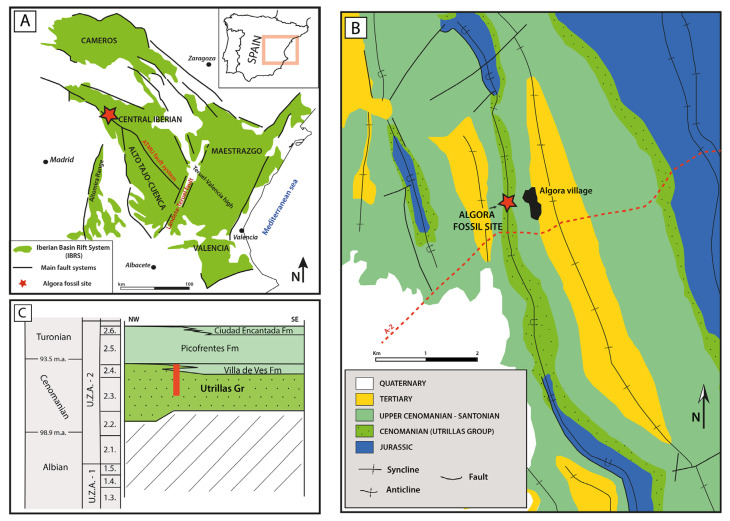
Geographical and geological location of the Cenomanian fossil site of Algora (Guadalajara, central Spain). (**A**) Geographical location of the Algora fossil site within the Iberian Basin Rift System and the Iberian Peninsula. Modified after [19]. (**B**) Simplified geological map of the Algora area, where the geographical location of the studied fossil site is shown. Based on previous data from [20]. (**C**) Chronostratigraphic scheme for the Albian–Turonian in the Ayllon–Siguenza Domain of the Iberian Basin Rift System. The vertical red bar indicates the position of the studied sedimentary section (see Figure 2). Modified after [16,21].

The studied plant macro-fossil assemblage was collected from the Cenomanian palaeontological area of Algora (Guadalajara Province, Central Spain), which is located 122 km northeast of Madrid city (Figure 1A). This area belongs to the Central Domain (i.e., the Ayllón-Sigüenza area) of the Iberian Basin Rift System (IBRS) [15] (Figure 1A). The IBRS, previously known as the Iberian Basin [16], is a Mesozoic intraplate rift basin of Iberia, extending across most of the eastern and northerncentral areas of Spain [15,17]. The IBRS was developed in relation to two episodes of rifting and their respective post-rift stages: the first episode spanned from the Permian to the latest Triassic [18], and the second from late–middle Jurassic to the latest early Cretaceous (late Albian) [15,17].

The sedimentary succession hosting the palaeontological outcrops of Algora is represented by the Utrillas Group and is included within the second post-rift stage, which encompasses the Upper Cretaceous (Figure 1B). The Utrillas Group [22], previously defined with the rank of formation [23], is a widely spread stratigraphic unit which presents a highly diachronic nature (spanning from the Albian to the Coniacian) across the IBRS and other basins of Iberia [24,25,26,27]).

**Figure 2:**
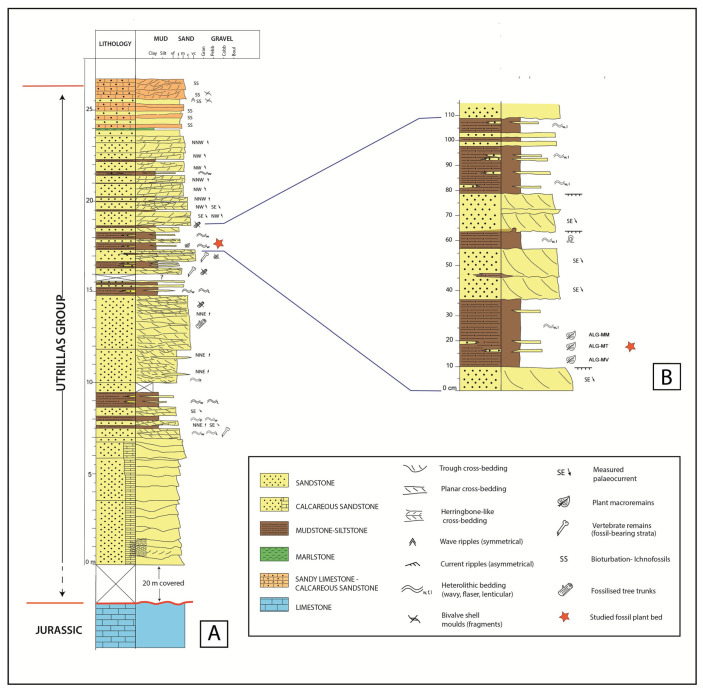
Sedimentary logged section of the Utrillas Group in the Algora area (Guadalajara, central Spain) (**A**), including a close-up section of the strata hosting the studied fossil plant beds (**B**).

In the study area, the Utrillas Group consists of an 80–100 m thick succession dominated by sandstones. The basal part of the unit is represented by alluvial deposits characterized by coarse-grained sandstones arranged into channelled bodies with erosional bases and silty-to-muddy floodplain deposits with local paleosols development. However, this lower part of the succession is covered in the surroundings of the Algora fossil site (Figure 2A).

Conversely, the sand bodies in the upper part of the Utrillas Group (where the macro-remains of vertebrates and fossil plants are located) are characterized by through and planar cross-bedding sets showing bipolar currents (herringbone-like strata), heterolithic laminations, and ferruginous crusts. The co-occurrence of these sedimentary structures indicates that the unit was deposited in inter- to subtidal sub-environments, characterized by estuarine bars and channels, confirming and providing solid sedimentological evidence for previous interpretations of an estuarine system [1,14]. In the surroundings of Algora, it overlays unconformably Jurassic strata [20] and, towards the SE, shifts laterally into the dolostones and limestones of the Villa de Ves Formation [28], deposited in peritidal environments (Figure 1C). In turn, the Utrillas Group and the Villa de Ves Formation are overlain by the uppermost Cenomanian–Lower Turonian Picofrentes Formation [29], which consists of open marine platform marls and limestones with ammonoids and nautiloids [16] (Figure 1C).

The plant macro-fossil assemblage described in this work was collected from the upper part of the coarse-grained sandstone level and from several thin beds at the top composed of an alternation of light brown to light green siltstones and heterolithic lamination, namely from base to top as ALG-MV, ALG-MT, and ALG-MM (Figure 2B).

In order to obtain biostratigraphic information by palynological data, rock samples were previously recovered from several locations within a thin layer composed of light grey to light green siltstones placed at the top of the series, but all the samples yield negative results (J.B. Diez, personal communication). Despite the lack of a proper biostratigraphic dating, the relative position of Algora within the upper part of the Utrillas Group allows one to infer a tentative middle Cenomanian–earliest late Cenomanian age [1,14] for the strata hosting the vertebrate remains [2,3,4,5,11,12,13], as well as the fossil plants presented in this work.

The studied material consists of 62 plant remains preserved as impressions, sometimes with carbonaceous or ferruginous patina (on several occasions preserved as a part and counter-part), but without preserved cuticles on leaves and stems or internal structures in the wood. Due the lack of cuticular characters, the fossils have been classified as a *sp.* in the generic level or as cf. referred to species. These fossils, corresponding to the specimens ALG 640 to ALG 701, are deposited in the Paleontological Museum of Castilla-La Mancha (MUPA), in Cuenca (Castilla-La Mancha, Spain).

## 3. Results—Systematic Paleontology

Pteridophyta

Order Filicales

Family *incertae sedis*

Genus *Pteris* Linnaeus

*Pteris* sp.

Figure 3A,B

Stratigraphic level: ALG-MT

Material: ALG 645

Description: Primary pinna 6 cm long and 4 cm wide between the tips of the lateral pinnae, rectangular in shape, with 13 to 14 secondary pinnae per lateral pinna arranged subopposite. Secondary pinnae 0.8 to 1.2 cm long and 3 mm wide, with subopposite pinnules. The pinnules are lanceolate, 1.7 mm long by 0.7 mm wide, with entire margins, acute apex, and venation consisting of a very thin central main vein extending to the apex. Secondary veins are not preserved.

Discussion: It resembles the leaves of the fern *Pteris* sp. from the middle Cenomanian of Nammoura, Lebanon in the size and morphology of both secondary pinnae and pinnules [30]. It could also be similar to some species of the genus *Cladophlebis* Brongniart, but venation is obscure in the Spanish fossil, and the secondary veins in the pinnules are not preserved.

**Figure 3:**
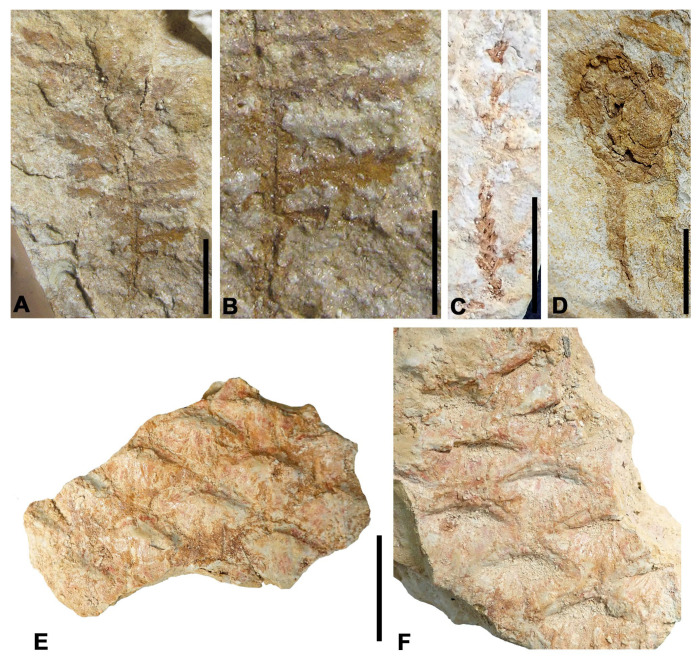
Ferns and conifers from the Algora site. (**A**) Pinna of *Pteris* sp. (**B**) Detail of pinnules in the lower right area of photo (**A**). (**C**) Axis of the conifer *Brachyphyllum* sp. (**D**) Conifer ovuliferous cone of *Conago* sp. with axis in connection. (**E**,**F**) Part and counter-part of the cone *Dammarites* cf. *albens* showing rhomboidal-shaped scales with transverse furrow. Scale bars: (**A**,**C**,**D**) 1 cm; (**B**) 5 mm; (**E**,**F**) 2 cm.

Gymnospermophyta

Order Coniferales

Genus *Brachyphyllum* Lindley et Hutton ex Brongniart emend. Harris

*Brachyphyllum* sp.

Figure 3C

Stratigraphic level: ALG-MT and ALG-MM

Material: ALG 646, ALG 699

Description: Fragments of simple axes between 1.4 and 2.2 cm long and 2 mm wide, bearing closely appressed rhomboidal-shaped leaves, 1.2 mm long and 0.8 mm wide on average, showing a central thickening along their entire length.

Discussion: The morphology of this genus consists of rhomboidal, scale-like leaves spirally arranged and closely appressed to the axis. The leaves are less than five times their width in length, have a broad basal cushion that narrows adapically, and the length of the free part or the total height of the leaf and cushion is as long as the width of the leaf cushion [31]. The leaves of this genus can be ascribed to different conifer families if the cuticle characters are preserved [31,32].

*Brachyphyllum* is a fossil genus of conifers widely distributed in Mesozoic assemblages worldwide that were previously reported from many Cenomanian coastal depositional environments opened to marine inputs in the Tethys realm [33,34,35].

Genus *Conago* Miller et Hickey

*Conago* sp.

Figure 3D

Stratigraphic level: ALG-MT

Material: ALG 658

Description: Sub-sphaerical-shaped cone, 1.3 cm in diameter, bearing rhomboidal to polygonal scales 4 mm in diameter, arranged in a helical pattern. The cone is connected at its base to a fragment of a conifer stem, 1.2 cm long and 2 mm wide, composed of apparently highly appressed, rhomboidal scale-like leaves 4 mm long and 2 mm wide.

Discussion: The genus *Conago* was erected by [36] to group ovuliferous cones of conifers of uncertain systematic affinity. The studied cone is similar to *Conago* sp. 2 from the middle Cenomanian Maletin Sandstone Formation of the Czech Republic [37] due to its general morphology, dimensions, and helically arranged rhomboidal scales.

Genus *Dammarites* C. Presl

*Dammarites cf*. *albens* C. Presl emend Hluštík

Figure 3E,F

Stratigraphic level: ALG-MT

Material: ALG 654

Description: Fragment (part and counter-part) of a large ovuliferous cone, 7 cm long and 6.5 cm wide, showing the impressions of the scale apices. These scales are rhomboidal to sub-elliptical in shape, 2 cm long and 1.3 cm wide, with entire rounded margins and a slightly pointed apex. The outer surface of the scales displays numerous parallel and fine veins extending in a fan-shaped pattern from the apex to the base. The apex has a rhomboidal protuberance, 7 mm long and 3 mm wide, located at its distal end, as well as a well-marked transverse groove running its entire width.

Discussion: It resembles the cone of the gymnosperm *Dammarites albens* C. Presl. emend Hluštík of the Peruc–Korycany Formation [38] from the middle–upper Cenomanian of the Czech Republic, based on its morphological characteristics and the arrangement and dimensions of its scales. According to some authors, this structure would correspond to an ovuliferous cone of a conifer related to the Family Araucariaceae, while others suggest it is a subterranean trunk of a cycad where the scales correspond to the bases of long ribbon-like leaves [38]. However, ref. [39] include it as an ovuliferous cone within the conifer group in their recent article on the flora and terrestrial environments of the Cenomanian of the Bohemian Basin in the Czech Republic.

Order Ginkgoales?

Genus *Pseudotorellia* Florin emend Kiritchkova et Nosova

*cf*. *Pseudotorellia* sp. 1

Figure 4A,B

Stratigraphic level: ALG-MT

Material: ALG 640, ALG 643, ALG 659

Description: Simple, lanceolate leaves, 2.7 cm long and 4 mm wide on average, with entire margins and a rounded-to-slightly-acute apex. Base not preserved. Venation consists of 7 to 8 thick, parallel veins running from the base of the leaf and converging near the apex.

Discussion: The most significant similarity in leaf morphology and venation is with the leaves of the supposed ginkgoalean genus *Pseudotorellia*, with specimens exhibiting similar characteristics found at the Nammoura site in Lebanon ([40] p. 40). However, a direct assignment to this taxon cannot be made since the leaves studied do not have a complete base, and the arrangement of the veins at origin cannot be known, so they have been classified as cf. *Pseudotorellia*.

**Figure 4:**
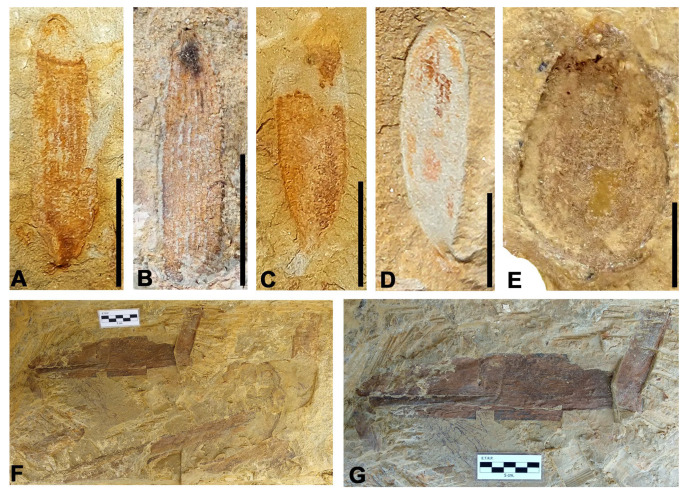
Gymnosperms from the Algora site. (**A**) An almost complete leaf of cf. *Pseudotorellia* sp. 1. (**B**) Central and apical part of a leaf of cf. *Pseudotorellia* sp. 1 showing gross parallel veins. (**C**,**D**) Complete leaves of *cf*. *Pseudotorellia* sp. 2 showing the short and curved petiole and numerous thin parallel veins. (**E**) Impression of indeterminate seed. (**F**) Several fragments of large leaves of *Desmiophyllum* sp. disposed in two apparent main orientations in the layer. (**G**) Detail of photo (**F**)showing venation of two fragments of large leaves of *Desmiophyllum* sp. Scale bars: (**A**,**C**,**D**,**E**) 5 mm; (**B**) 1 cm; (**F**,**G**) 5 cm.

cf. *Pseudotorellia* sp. 2

Figure 4C,D

Stratigraphic levels: ALG-MV, ALG-MM

Material: ALG 651, ALG 652, ALG 655, ALG 676, ALG 693, ALG 694, ALG 701

Description: Simple leaves, elliptic to lanceolate-shaped, between 1.2 cm long and 3 mm wide on average, with entire margins, rounded apex, and an acute base. They show a petiole that is short and straight, up to 2 mm long, or sometimes curved and continuous with the leaf base. The venation consists of at least 12 fine veins arranged parallel to each other, running from a central point at the base and converging near the apex.

Discussion: The leaves studied have a morphology similar to that of the conifer *Elatocladus* Halle, but these have a very prominent midrib which is not found in the Algora specimens. Leaves with the same characteristics as those from Algora were classified as belonging to *Pseudotorellia* sp. in specimens from the Nammoura site in Lebanon [40] p. 40.

Order *incertae sedis*

Gymnospermous seed

Figure 4E

Stratigraphic level: ALG-MM

Material: ALG 653

Description: Seed with a sub-conical morphology and rounded triangular cross-section, 1.5 cm long and 9 mm wide at its widest point, with a rounded base and pointed apex and preserved as a concave internal mould. It shows a well-defined and thin outer layer, an intermediate layer 1.5 mm thick, an internal cavity 1 cm long and 6.5 mm wide, and what appears to be the microphyll, subtriangular in morphology 2 mm long.

Discussion: The morphology and internal structure resemble those of extant cycad seeds, although it could also correspond to seeds of other gymnosperms, such as ginkgoales or some types of angiosperms.

Genus *Desmiophyllum* Lesquereux emend Miller et Hickey

*Desmiophyllum* sp.

**Figure 5:**
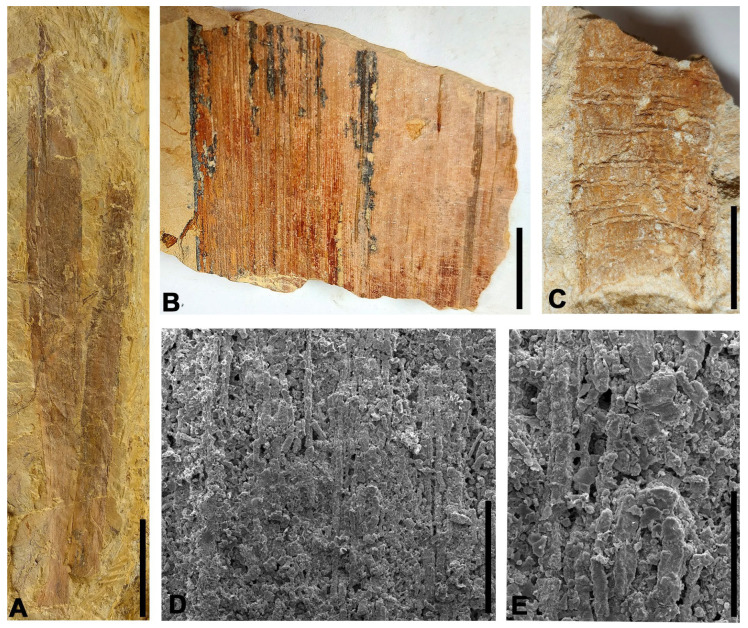
*Desmiophyllum* sp. leaves from the Algora site. (**A**) Two large leaves, with the left leaf complete. (**B**) Detail of venation in the counter-part of the left leaf in photo (**A**). (**C**) Impression of stem assigned to the genus *Desmiophyllum* showing parallel linear-shaped leaf base scars. (**D**) SEM image showing micro-veins between main ones. (**E**) Detail of several micro-veins in photo (**D**). Scale bars: (**A**) 10 cm; (**B**) 1 cm; (**C**) 2 cm; (**D**) 200 µm; (**E**) 100 µm.

Figure 4F,G and Figure 5

Stratigraphic levels: ALG-MT, ALG-MM

Material: leaves ALG 647, ALG 657, ALG 661-ALG 675, ALG 677-ALG 692, ALG 695-ALG 700; stems: ALG 656, ALG 660

Description: Large fragments and one complete specimen of very large strap-shaped parallel-veined leaves with acute apex and leathery appearance. The complete leaf is 62 cm long and 5.8 cm in its maximal width, but fragments of other leaves are up to 8.2 cm wide. The margins are entire, very well-marked, and remain parallel to sub-parallel throughout the leaf, but converge at the apex. Base straight to slightly curved and apex acute. The venation consists of a set of parallel thick veins, with more than 170 veins in the leaf (44 veins per cm on average in its widest part), running independently from the basal zone and converging at the apex. Separation between veins is 0.2 mm. One of the leaves exhibits a microstructure observable with SEM, composed of parallel, straight micro-veins with irregular walls arranged between the main veins, with approximately 40 micro-veins per mm.

Associated with these ribbon-like leaves, two impressions of cylindrical-shaped stem fragments with preserved transverse marks were found in the ALG-MT level. These marks appear to correspond to basal insertion scars of leaves with very broad, laminar bases. The best-preserved stem is 5.6 cm long and 2.8 cm wide, and it has up to 12 linear transverse scars, 1.5 mm thick, with sinuous margins. These scars are laterally wedged, do not span the entire width of the stem, and are arranged parallel to each other with a spacing of 2 to 2.5 mm.

Discussion: The genus *Desmiophyllum* Lesquereux was formally emended by [36] to classify the leaves of Mesozoic gymnosperms with elongated ribbon morphology and parallel, very profuse venation, but which do not present defined taxonomic characters to include them in a specific family. Leaves of genus *Dammarites* C. Presl from the Cenomanian of Central Europe show many similarities to *Desmiophyllum*, and, as indicated by [38,41], the former name should have priority. Nevertheless, we have classified the specimens from Spain as *Desmiophyllum* in the sense of [36] because the studied leaves are isolated, and *Dammarites* must be used if the leaves are in connection to the ovuliferous cone where they were originally found [38,42].

Leaves having similar morphologies are recognizable in other groups such as cycadophytes and monocotyledonous angiosperms. Nevertheless, venation in the studied leaves is much more profuse than in segments from leaves of cycads and bennettitales, and leaves of monocots have short transversal veins between the mains that are not present in the Spanish specimens [43].

Regarding leaf morphology, the leaves from Algora resemble those from the Albian–Cenomanian boundary at the Mosqueruela locality in the Teruel region, northeastern Spain [44], in terms of the number of veins per cm and the absence of macro-interveins between the main ones.

In addition, the micro-characteristics visible under SEM of one of the fossils from Algora resemble those from ribbon-like strap-shaped leaves classified as *Phragmites* from the middle–late Cenomanian of Nammoura, Lebanon [45]), in the number of micro-parallel veins between main ones, that is, 30 to 40 veins per mm, similar to the 44 veins per mm in the Spanish fossil. This fossil from Nammoura was originally assigned to monocotyledons angiosperms due to its venation pattern composed of numerous parallel veins [45]. Nevertheless, due to the Lebanese specimen lacking the characteristic transverse veins between the main veins, it would be assigned to the indeterminate gymnospermous genus *Desmiophyllum*.

Angiospermophyta

Angiosperm type 1

Figure 6A,C

Stratigraphic level: ALG-MT

Material: ALG 641, ALG 644

Description: Leaves simple with a blade 3.8 cm long and 2 cm wide at its widest point. They are obovate-shaped with entire margins, although the apex is slightly crenulated. Base cuneate and apex acute. The leaves have a broad, well-marked midrib 1 mm wide and secondary veins arranged in an apparently craspedodromous pattern, arising from the midrib at approximately 45°. The leaves have a petiole that in some cases can reach 1.4 cm in length and 1.5 mm in width, which is inserted directly at the base.

Discussion: This leaf resembles those of *Diospyros cretacea* Velenowsky et Viniklář, from the Peruc–Korycany Formation of the middle–late Cenomanian of the Czech Republic [39] in the morphology of the blade and in the attachment of the petiole in the leaf. It also resembles the leaves of *Ascarinophyllum pecinovense* Čepičková et Kvaček in the morphology of the blade and in having a serrated margin at the apex area. However, our specimens lack a serrated margin in the central area of the blade, unlike the Czech specimens that present this feature [46].

Angiosperm type 2

Figure 6B,D

Stratigraphic level: ALG-MT

Material: ALG 642

Description: Leaf fragment, 4 cm long and 1.5 cm wide at its maximum preserved width, lanceolate in shape, widening towards the apex and tapering towards the base. Leaf margins entire, and base and apex not preserved. Venation consists of a single broad central vein and craspedodromous secondary veins at nearly 60° from the main vein. The leaf blade is perforated due to insect feeding interactions, with elliptical-shaped elongated perforations, measuring 11 mm long and 4 mm wide at its maximum width, wavy-to-sinuous margins and a poorly defined reaction margin.

Discussion: It resembles the leaves of the lauraceous angiosperm *Myrtophyllum geinitzii* Heer of the Peruc–Korycany Formation, from the middle–late Cenomanian of the Czech Republic in its morphology and craspedodromous secondary venation [39].

The insect interactions on the leaf blade are consistent in morphology and dimensions with hole-feeding damage of type DT07, as was defined in the insect damage classification of [47].

Angiosperm type 3

Figure 6E–G

Stratigraphic level: ALG-MT

Material: ALG 648, ALG 649

Description: Leaf fragments up to 6.4 cm long and 1 cm wide, with a narrow lanceolate-to-fusiform morphology and a narrow cuneate base. Apex not preserved. The margins are apparently entire, and the venation consists of a well-marked midrib and bilateral intramarginal veins arranged subopposite that merge into narrow acute angles and run parallel to the margin. Secondary venation not preserved.

Discussion: They resemble the leaves of *Myrtophyllum geinitzii* Heer from the middle Cenomanian Maletin Sandstone Formation of the Czech Republic [37] in terms of general morphology. They also resemble the leaves of the genus *Eucalyptolaurus* Coiffard, Gomez, Thiébaut and Kvaček from the middle–upper Cenomanian of the Czech Republic [39] and France [48] in the morphology of the leaves, the entire margins, and the presence of lateral veins that run along the leaf from the base.

**Figure 6:**
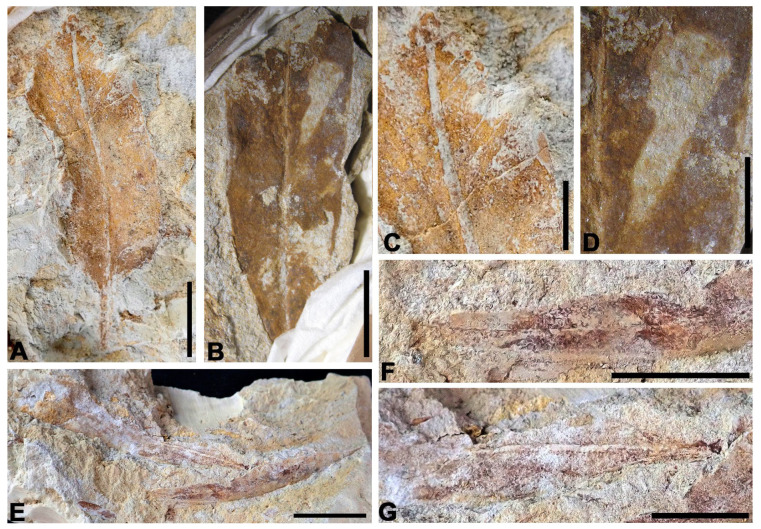
Angiosperms from the Algora site. (**A**) Angiosperm type 1. (**B**) Angiosperm type 2. (**C**) Detail of apex in Angiosperm type 1. (**D**) Detail of one of the plant–insect interactions in Angiosperm type 2. (**E**) Angiosperms type 3. (**F**) Detail of the middle part of lower leaf in photo (**E**). (**G**) Detail of the base of the upper leaf in photo (**E**). Scale bars: (**A**,**B**,**F**,**G**) 1 cm; (**C**,**D**) 5 mm; (**E**) 2 cm.

## 4. Discussion

### 4.1. Paleobiogeographical Discussion

The fossil record in the Algora site sheds light on the poorly known transition between the Lower and the Upper Cretaceous vertebrate faunas of Europe ([1] and references therein). Although the fauna identified at this Cenomanian locality includes some lineages well-established in Lower Cretaceous European ecosystems (e.g., helochelydrid turtles), many of the identified taxa are of Gondwanan origin (e.g., bothremydid turtles, abelisaurs, and lithostrotians). The dispersal of these faunas to Laurasia was very successful, replacing those of the Lower Cretaceous. Algora represents some of the most abundant and diverse continental vertebrates in the Upper Cretaceous terrestrial ecosystems of Southern Europe [1].

Regarding the Algora macro-paleobotanical record, it represents a significant contribution to the knowledge of the plant assemblages of the middle–late Cenomanian in the western and central Tethysian realm, as the different floras identified in this area, corresponding to this time span, show clear differences depending on both their paleogeographical locations and their associated coastal environments. In this respect, the Central European macro-floristic records from Cenomanian coastal environments present different macro-fossil assemblages comparable to those of the Algora site. Thus, in France, numerous Cenomanian-age sites contain plant assemblages that differ from or partially overlap with the Algora flora. The coastal marshland assemblages of the Cenomanian of Anjou [49] and Archingeay–Les Nouillers [50] contain a high abundance of ginkgoalean leaves of the genus *Erethmophyllum* Thomas and conifer stems of the genus *Frenelopsis* Schenk, as well as axes of the genus *Glenrosa* Watson et Fisher in the latter site. Floral assemblages in coastal marine and lagoon environments composed primarily of xerophytic conifer stems of the genera *Frenelopsis* and axes of *Glenrosa*, as well as axes of the genus *Geinitzia* Endilcher, are also present in the lower Cenomanian of Archingeay–Les Nouillers [35,51], Monragne [52], and Châtellerault [53], but these taxa have not been identified in the Algora assemblage.

Also in France, other macro-remain assemblages show certain similarities to the Algora flora, such as the lower Cenomanian coastal marshland assemblage at La Gripperie–Saint-Symphorien, Charente-Maritime [54], which contains several types of matoniaceous ferns as well as conifers of the genera *Geinitzia* and *Pagiophyllum* Heer that do not occur at Algora, but also contains angiosperm leaves including cf. *Eucalyptolaurus*, which are recorded at the Spanish site. Among these French Cenomanian assemblages, the most similar in composition to the Algora assemblage is the lower Cenomanian coastal freshwater assemblage with marine inputs from Jaunay-Clan [55]. This diverse plant community contains ferns of the genus *Cladophlebis* as well as conifers of the genera *Brachyphyllum* and *Pagiophyllum*, the gymnosperm genus *Dammarophyllum,* and leaves of terrestrial angiosperms of the genus *Eucalyptolaurus*, among others.

The middle–upper Cenomanian deposits of the Czech Republic exhibit several environmental assemblages related to fluvial, floodplain, fire-affected savanna, fluvio-coastal, and brackish marsh deposits [39] and publications therein. The fluvial–coastal estuarine assemblage in this area shows the most significant similarity to the Algora assemblage, as it presents large, parallel-veined strap-like leaves and large cones assigned to *Dammarites albens*, as well as various types of conifers such as *Brachyphyllum* and *Conago,* ferns, and angiosperm leaves, some of which, such as *Myrtophyllum, Eucalyptolaurus, Diospyros cretacea,* and *Ascarinophyllum pecinovense*, resemble specimens found at the Spanish site.

Regarding the macro-floristic records from Cenomanian coastal environments in North Africa, there are several fossil sites containing macro-floral assemblages sharing some taxa with the Algora flora. This is the case of the very long ribbon-shaped leaves from the Cenomanian Akrabou Formation in the Gara Sbaa locality in Morocco [56] and the Merbah el Asfer site in Tunisia [57]. These leaves were classified within the genus *Welwitschiophyllum*, which has been linked to the gnetales group [56,58,59] but shares many similarities with the leaves of *Desmiophyllum* from Algora. However, the specimens from North Africa show short transverse veins between the main ones, a feature that the Spanish leaves lack, thereby excluding them from the genus *Welwitschiophyllum*.

Another important location containing an early Cenomanian macro-floral assemblage in fluvial, coastal, and near-shore deposits of North Africa is the Bahariya Formation in the Bahariya area and nearby locations in Egypt. This flora is dominated by a variety of broad-leaved angiosperms, including both terrestrial and aquatic elements, and some ferns and gymnosperms, including ribbon-shaped parallel-veined leaves similar to those from Algora [60,61]. This geological formation also presents evidence of charcoalified conifer wood fragments [60,62], in contrast to the Algora site, where there is no evidence of charcoalified records to date.

The location in the Middle East containing the most comparable assemblage with Algora is the site where most fossil plants from Lebanon were found. It is the famous early-middle Cenomanian fossil site of Nammoura, where twelve species of ferns, such as *Pteris* sp., gymnosperms, including conifers as *Brachyphyllum*, putative ginkgophytes, such as *Psudotorellia* and other cryptic fossils such as the long ribbon-like parallel-veined leaves of *Phragmites* and also several taxa of angiosperms, have been described [30,45,63]. According to recent research, this assemblage is very different from the Cenomanian floras of other Middle Eastern and North-African fossil sites, but is more closely related to coeval floras found in contemporaneous Central European sites [30,45,64].

### 4.2. Taphonomical (Biostratinomical) and Paleoenvironmental Discussion

All plant remains found so far at the Algora site belong to terrestrial plants, indicating an input from emerged areas. This input would have occurred through the fluvial and/or fluvio-tidal channels developed in the area, in addition to gravity input at some levels from the shoreline of the depositional environment.

The absence of open marine fossils in any of the layers of the site containing plant remains suggests that the coastal beach or littoral environments where the remains are found were protected from direct marine input by some type of natural barrier.

**Figure 7:**
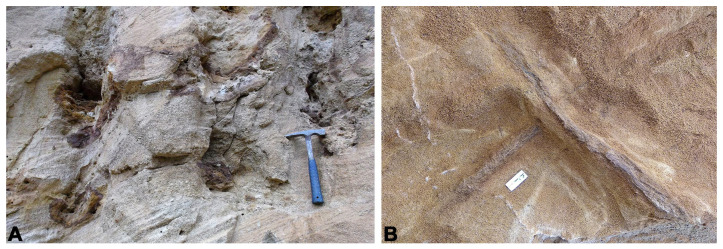
(**A**) Natural ferruginous casts of fossil trunks in sandstone bodies located at the base of the series in the Algora fossil site (metres 12–14 of the logged section; Figure 2), some of them probably preserving part of the roots in connection. (**B**) Two fragments of ferruginous wood disposed in perpendicular orientations at the top of the series (approximately at metre 18 of the logged section; see Figure 2) containing the vertebrate remains in the Algora site.

The channel-like sandstone levels at the base of the site contain concentrations of tree trunks ranging in size from very large (metre-sized), some with what could be part of the root bases, to medium and small centimetre-sized, all apparently oriented (Figure 7A). These trunks were carried and concentrated by water currents and accumulated in high-energy channels and bars. The place where these plants grew would not be very far from their depositional area since some of the trunks retain part of the root bases. The depositional environment of this stratigraphic level could be consistent with fluvio-tidal channel deposits and proximal floodplain in a near-shore environment.

The stratigraphic level bearing the main concentration of vertebrate remains at the Algora vertebrate site is located in layers of coarse-grained sandstone, in which some faunal remains are fragmented and isolated as dinosaur bones, teeth and crocodile osteoderms, and turtle plates. However, partial and even relatively complete and complete turtle carapaces are very abundant, corresponding to individuals of *Algorachelus peregrina* at different ontogenetic stages. The plant remains in this layer consist of medium-sized, ferruginized fragments of branches and trunks, ranging from centimetric to decimetric in length and centimetric in width. These fragments are scarce, preserving rounded and/or eroded external contours, and exhibit varying inclinations within the stratum, lacking a preferential orientation (Figure 7B). These remains correspond to fragments of detached xylem carried by high-energy water currents that were repeatedly eroded by remobilization within the sedimentary environment.

In some cases, they may have remained in the aquatic environment for a prolonged period, as evidenced by their inflated surfaces, which form rounded-to-ellipsoidal domes due to water saturation. The depositional environment of this stratigraphic level would be consistent with an abandoned tidal depression or channel in a near-shore area, with sporadic contributions from other channels or periodic flooding.

The ALG-MV stratigraphic level contains very small, highly fragmented, and scattered plant remains, including a few complete leaves of cf. *Pseudotorellia* type 2, indicating that the remains underwent high-to-moderate energy transport, carried by flotation from relatively long distances. The depositional environment of this stratigraphic level would be consistent with an inter-to-subtidal zone of an estuarine system in which sandy tidal channels and creeks cut heterolithic tidal flat deposits.

The ALG-MT stratigraphic level contains medium-sized plant remains, some of which were transported from areas close to the depositional zone. These remains are found with their parts still connected, such as a primary pinna with secondary pinnae and pinnules attached to their rachises and neither broken nor bent; angiosperm leaves with petioles; or conifer axis connected to an ovuliferous cone. However, other plant remains may have undergone more prolonged transport under moderate-energy conditions, as evidenced by their fragmented state, such as segmented tree trunks and fragments of broad angiosperm leaves with broken blades. The depositional environment of this stratigraphic level would be consistent with an inter-to-subtidal zone of an estuarine system in which sandy tidal channels and creeks cut heterolithic tidal flat deposits, yet most likely in a more proximal position than that of the level ALG-MV.

The ALG-MM stratigraphic level shows a massive accumulation of very long and wide strap-shaped leaf fragments of genus *Desmiophyllum*, sometimes complete leaves, in massive concentrations (Figure 4F,G and Figure 5A) with an almost mono-specific association where only a few leaves of cf. *Pseudotorellia* type 1 also occur. The plant remains in this stratigraphic level underwent minimal transport from their production area, as some long, strap-shaped leaves with parallel venation of the genus *Desmiophyllum* preserve the margins, base, and apex intact. Actuotaphonomical studies indicate that leaves of this type usually undergo shredded fraying of the blade in favour of the veins when they fall into an aqueous environment and are then transported with a certain amount of energy [65]. The depositional environment of this stratigraphic level would be consistent with a pond between tidal channels or a mudflat in the upper intertidal zone of an estuarine system.

In relation to the environmental conditions under which the plants of Algora could have grown, it is noteworthy that many of the plants from the coeval Nammoura site in Lebanon, which also occur in Algora, are considered xeromorphic. This fact implies adaptation to grow under drought conditions, which could have been similar to those of the present-day Mediterranean climate [30,45,63].

It is also noticeable that the record of plant–insect interactions from angiosperm leaves at Algora indicates the presence of insects in coastal environments of this area of the Iberian Plate, thereby increasing the diversity of organisms that roamed this area of the Tethys realm during the middle–late Cenomanian.

## 5. Conclusions

The macro-floral assemblage from the vertebrate fossil site of Algora (Guadalajara, Central Spain) shows diversity of taxa, including ferns, conifers, possibly ginkgoales, cryptic gymnosperms, and angiosperms that lived on the shores of the fluvial–estuarine coastal system developed during the middle–late Cenomanian in this area of the westernmost part of the Tethysian realm. This assemblage, analyzed here for the first time, shows similarities with other coeval ones from the Tethys realm, particularly with the floras of the Czech Republic and with the flora from Nammoura in Lebanon. The biostratinomical study of the fossil plants from the Algora site indicates some variability in the environments, as suggested by the occurrence of different types of plants, where the almost mono-specific assemblage of *Desmiophyllum* leaves indicates a possible para-autochtony of the plant producers in low-energy environments within the fluvial–coastal system.

## Data Availability

The original contributions presented in this study are included in the article. Further inquiries can be directed to the corresponding author(s).
